# Impaired Spatial Category Representations in Williams Syndrome; an Investigation of the Mechanistic Contributions of Non-verbal Cognition and Spatial Language Performance

**DOI:** 10.3389/fpsyg.2016.01868

**Published:** 2016-11-28

**Authors:** Emily K. Farran, Lauren Atkinson, Hannah Broadbent

**Affiliations:** ^1^Department of Psychology and Human Development, UCL Institute of Education, University College LondonLondon, UK; ^2^Centre for Behaviour and Child Development, Birkbeck, University of LondonLondon, UK

**Keywords:** Williams syndrome, neurodevelopmental disorder, space, spatial categories, spatial language

## Abstract

The aims of this study were to: provide a precise characterisation of spatial category representations in Williams syndrome (WS); to determine the nature of the mechanistic contributions from spatial language performance and non-verbal cognition to spatial category representations in WS; and to explore the stability of spatial category representations in WS using error analysis. Spatial category representation was assessed across nine spatial categories (In, On, Under, In Front, Behind, Above, Below, Left, and Right) using an odd-one-out task. The performance of individuals with WS (*N* = 24; 12;00 years;months to 30;07 years;months) was compared to data from typically developing children aged four to 7 years (*N* = 75), published in Farran and Atkinson (2016). The WS group performed at the level of typical 4- and 5-year-olds. Despite this low level of ability, they demonstrated typical variation in their representation of easier to harder spatial categories, in line with the spatial category representation model (Farran and Atkinson, 2016). Error analysis of broad category understanding (i.e., category understanding which includes non-prototypical category members), however, showed that errors reflected fewer guess responses than expected by chance in the WS group only, which could suggest strategic responding in this group. Developmental trajectory analyses demonstrated a significant contributing influence of both non-verbal mental age and spatial language ability in the TD group. For the WS group, non-verbal mental age significantly contributed to spatial category representations, whilst the contributing influence of spatial language ability was marginally significant. With reference to level of ability, spatial category representations in the WS group were consistently lower than would be expected for non-verbal mental age, but on a par with their (low) spatial language mental age. Spatial category representations in WS are discussed with reference to their contribution to the hallmark deficit in spatial construction and drawing abilities in WS.

## Introduction

Williams syndrome (WS) is a neurodevelopmental disorder with a prevalence of one in 7,500 to one in 20,000 (Morris and Mervis, 1999; Strømme et al., 2002). As a group, a defining cognitive characteristic of WS is the discrepancy between poor spatial cognition compared to relatively strong verbal cognition. Whilst most spatial skills do not develop beyond the level of a typically developing (TD) 6-year-old, individuals with WS demonstrate an uneven profile of spatial performance (Farran and Formby, 2012). A profound deficit in performance on visuo-spatial construction tasks, such as the Wechsler Block Design task (e.g., Wechsler, 1992), is considered a hallmark of WS (Mervis et al., 1999). Performance on this task does not usually develop beyond the level of a TD 4-year-old (Farran and Formby, 2012).

Success on visuo-spatial construction and drawing tasks rests on the ability to perceive the spatial relationships among the parts of the to-be-copied image, and to recreate the same spatial relationships in the copied image. In order to encode the spatial relationships among objects or parts of objects one must ascertain the position of one object relative to another. This can be represented as a spatial category (e.g., *left* of, *above*). Performance on visuo-spatial construction and drawing tasks in WS suggests an underlying deficit in perceiving and producing spatial relationships. This is evidenced in a number of ways. First, performance is characterized by a lack of global cohesion when producing the model image. That is, participants often produce disjointed responses in which the spatial relationships among the individual blocks, or parts of a drawing, are incorrect (e.g., Bellugi et al., 1988). Second, analytical studies of the errors made on block construction tasks have shown that participants with WS do not discriminate between mirror imaged block faces. For example, participants with WS confuse a block face with white above red, with the opposite arrangement of red above white (Hoffman et al., 2003; Farran and Jarrold, 2004). This is again indicative of impoverished spatial relationship understanding. Finally, people with WS make fewer checks of their part-finished solutions during block construction and drawing tasks than do TD controls (Hoffman et al., 2003; Hudson and Farran, 2013). This suggests attenuated attention to, or a lack of ability to adhere to, the spatial relationships of the model image.

Few studies have addressed spatial relationship understanding in WS directly. Farran and Jarrold (2005) specifically asked participants with WS to categorize the spatial relationship between a bat and a ball (for example, the ball is *above* vs. *below* the bat). Performance was not only poor, but showed evidence that spatial category boundaries (e.g., the dividing boundary between the category of *above* and *below*) were atypical in the WS group, relative to TD controls. This fragility in category boundaries could influence the extent to which non-prototypical category members are considered members of that spatial category. For example, the extent to which *diagonally above* is categorized in the category of *above*. Furthermore, Landau and Hoffman (2005) demonstrated that spatial category representations of *above, below, left*, and *right* were less well-defined in WS than mental age (MA) matched TD controls. The WS group demonstrated typical category representations for objects that were prototypes of the spatial categories (i.e., the object was located on an extension of the referent’s axis and close to the referent). In contrast, the WS group showed deficits relative to the control group as the distance between the object and referent increased, for both on-axis and off-axis category examples, i.e., for non-prototypical category members. The results of these two studies suggest that whilst individuals with WS do have some understanding of spatial relationships and how these are categorized, this understanding is fragile. The predominant aim of the current study is to explore the representation of spatial categories further, by assessing the representation of nine spatial categories in WS. This will enable us to determine whether the evidence for impaired spatial category representations in WS discussed above, extends beyond *above, below, left*, and *right.*

We will accomplish our aim by examining spatial category representations in WS with reference to the spatial category representation model (Farran and Atkinson, 2016), described below. This will enable us to determine to extent to which the development of spatial category representations are typical or atypical in WS. The spatial category representation model describes the development of spatial categories across three levels. In the typical population, during the pre-school and primary school years, children demonstrate mastery of each successive level with increasing age. Level 1, rigid understanding, refers to a rigid understanding of spatial categories in which an understanding of a ball *on* a table is difficult to transfer to other object-referent pairs such as a car *on* a box. Level 2, abstract understanding, refers to the ability to recognize common spatial relations across multiple object-referent pairings (the transition from rigid to abstract spatial category representation is based on Casasola, 2008). Level 3, broad understanding, involves the use of a prototype framework (Erreich and Valian, 1979; Meints et al., 2002). Once a child reaches level 3 understanding, in contrast to levels 1 and 2, they now include both prototypical and non-prototypical examples as category members. That is, level 3 relies on children understanding broad category membership. Using this model, we have demonstrated that TD children progress through these three levels of understanding as their spatial category representations develop. For example, for the category *above*, evidence of level 1 understanding was shown at 4 years, level 2 understanding at 5 years, and level 3 understanding at 7 years. This progressive developmental pattern was common to all nine spatial categories investigated. Furthermore, the age at which children progressed from one level to the next varied across categories, broadly in line with predictions based on the emergence of spatial category representations in infancy. For example, level 3 understanding was evidenced for *in* and *under* earlier than *in front* and *behind* (Farran and Atkinson, 2016). We predict that spatial category representations will be impaired in WS, perhaps not progressing fully to level 3 (broad category membership) understanding for some spatial categories. Evidence for narrow category membership in WS with reference to the categories of *above* and *below*, *left* and *right*, supports this prediction (Landau and Hoffman, 2005).

Spatial category representations are inextricably linked to spatial language. Discussion now turns to the association between spatial category representations and spatial language within the context of both typical development and WS. Evidence from both TD infants (Casasola, 2008; Balcomb et al., 2011) and TD primary school children (Farran and Atkinson, 2016) demonstrates that spatial category representations are yoked to the verbal labels used to describe those categories. This could reflect the use of verbal labels for spatial categories, but equally could reflect spatial language experience. That is, children gain increasing knowledge that verbal labels can be used to discriminate objects and to group them together, and that this has an impact on how they attend to and categorize objects. The relative difference between weaker spatial vs. stronger verbal competence in WS questions the extent to which these two domains interact with reference to spatial category representations in this group. Landau and Hoffman (2005) asked this question directly. They assessed spatial language use in their WS group and in TD controls, for comparison to their spatial category representation task described above. They demonstrated that TD 5-year-olds applied the terms *above*, *below*, *left*, and *right* more readily to objects that were directly on horizontal or vertical axes (prototypical category members), than to objects that were slightly off axes (non-prototypical category members). However, they were equally able to use these terms for objects near to the target (prototypical category members) or far from the target (non-prototypical category members). Children with WS showed typical directional errors between *above* and *below*, *left*, and *right*, and a typical preference for on-axis locations. In contrast, unlike the TD controls, the children with WS were more likely to produce the words *above* and *below* for near than for far objects. This mirrors the pattern of results, and the group differences evident in their non-linguistic task, described earlier, suggesting that spatial language interacts with spatial representation in WS, as it does in TD children.

Studies that have focussed solely on spatial language in WS also show impaired performance. A deficit in spatial language in WS could reflect impairment in encoding spatial categories within the non-linguistic spatial domain (e.g., Bowerman, 1996). Phillips et al. (2004) designed the Test for Receptive Understanding of Spatial Terms (TRUST) to explore spatial comparative terms in individuals with WS. In this test, children hear a sentence that includes a spatial comparative such as “The dog is *in front* of the house,” and are asked to point to the picture that corresponds to the sentence, from a set of four. Phillips et al. (2004) found that individuals with WS struggled to understand these spatial terms, relative to TD controls and individuals with moderate learning difficulties of the same verbal MA. Relatedly, Heinze et al. (2014) demonstrated that spatial relational vocabulary was marginally weaker than concrete vocabulary in WS. These findings were further supported by Laing and Jarrold (2007) who demonstrated that individuals with WS find it difficult to create a mental model of spatial language terms. They used the same sentences for two tasks, for example: “The red animal is *taller* than the green animal.” The tasks were a semantic picture matching task and a spatial picture matching task. In both tasks, the participant was asked to point to one of four images in response to hearing the spatial sentence. In the semantic picture matching task the two animals presented were physically the same size and participants had to rely on their semantic knowledge of the animals in the real world (i.e., which is *taller*, a giraffe or a penguin?). In contrast, in the spatial picture matching task animals were presented as physically different sizes and participants had to make judgements related to the physical presentation of the animals. Laing and Jarrold (2007) found that whilst WS performance was poor on both tasks, it was significantly poorer on the spatial picture matching task than the semantic picture matching task, relative to controls. They suggest that this can be explained by the additional demand of mapping the verbal description onto the spatial image. Thus, it is not just the semantics of spatial language that presents a difficulty in WS, but the demands of creating a spatial mental model of the verbal description in order to map this onto the possible pairs of animals shown. This suggests that the yoking between spatial and language domains in WS might be compromised in some situations. A further aim of the current study, therefore, is to determine the associations between spatial category representations and the comprehension and production of the verbal labels for those categories in our WS group, for comparison with typical development. Whilst our measure of spatial category representations (the odd-one-out task) is deliberately non-verbal, it is possible that participants will use verbal coding when categorizing images.

In summary, individuals with WS have a profound deficit in visuo-spatial construction and drawing. Despite this, few studies have addressed the understanding of spatial relationships and spatial category representation in WS, which is arguably a key mechanism for success on such tasks. Where studies have focussed on spatial relationship understanding in WS, impairment is reported, with some evidence of atypical features (e.g., Farran and Jarrold, 2005; Landau and Hoffman, 2005). In this study, we used our odd-one-out task, previously developed for primary school age children (Farran and Atkinson, 2016) to assess spatial representations among individuals with WS for nine spatial categories: *In, On, Under, In Front, Behind, Above, Below, Left*, and *Right.* The data from these individuals with WS was compared to that previously reported for 75 TD children, aged 4–7 years, on whom the spatial category representation model was validated (Farran and Atkinson, 2016). Here, we use these TD data as a reference against which to explore the patterns of performance of the WS group and the developmental trajectory of WS spatial category representation formation.

Previous findings point toward narrow category membership in WS for the categories of *above*, *below*, *left*, and *right* (Landau and Hoffman, 2005). This predicts a particular impairment in reaching level 3 of the spatial category representation model. In light of this, we also conducted an error analysis of level 3 performance, for comparison between WS and TD groups to gain insight into strategy use at level 3. This is a novel analysis to both the TD and WS groups. Given the paucity of research on spatial category representations in either TD primary school age children or individuals with WS, it is difficult to predict whether errors will demonstrate strategic responding as opposed to guessing. If they do, it is likely that these will be developmentally linked in the TD group such that strategic responding increases with age. For the WS group, if spatial category representations are delayed and not deviant, the error analysis will also demonstrate typical patterns of errors, but at a level commensurate with a younger TD child.

In this study, the participants have already acquired language and so we do not aim to speak to the direction of influence between space and language domains; we highlight that Balcomb et al. (2011) consider there to be no clear evidence of a one-directional influence between language and space. Rather, we predict that spatial category representations will be impaired among individuals with WS in line with their other spatial cognitive abilities. By comparing WS performance to a large age range of TD children, we will also be able to determine the extent to which spatial category representations represent deviant development or delayed development. We also predict that spatial language will be poor in WS, and thus will be impaired relative to each individuals’ general verbal ability. This would demonstrate the plasticity of the developing brain, such that a deficit in the spatial domain can influence the development of the verbal domain. The relationship between spatial category representations and spatial language in WS is difficult to predict. On the one hand, based on Laing and Jarrold (2007), spatial category representations might not be spontaneously verbally labeled due to a difficulty mapping a spatial mental model of the category onto linguistic terms. On the other hand, Landau and Hoffman (2005) report similar patterns of performance on non-linguistic and linguistic spatial category tasks in WS, suggestive of an association between spatial and language domains. This is supported by studies that have demonstrated that verbal mediation is effective in WS when tackling spatial tasks (e.g., Farran et al., 1999, 2010). Clearly, the limited knowledge in relation to spatial category representation in WS, and the mixed pattern of findings in relation to the cross-domain relationship between spatial language and spatial cognition in WS indicate that the current study is warranted.

In light of the above, the aims of the current study are as follows. First, to characterize spatial category representations in WS across nine spatial categories with reference to the spatial category representation model (Farran and Atkinson, 2016). Second, to explore the extent to which (a) spatial language performance and (b) non-verbal cognitive ability, contribute to spatial category representations in WS. Finally, when Level 3 broad category understanding is fragile, we aim to gain insight in to the nature of participants’ representations by analyzing the patterns of error responses across groups.

## Materials and Methods

### Ethics

This study abides by the British Psychological Society (BPS) Code of Human Research Ethics and Code of Ethics and Conduct. Ethical approval for this study was obtained from the UCL Institute of Education ethics committee.

### Participants

Twenty-four participants with WS aged between 12;00 years;months and 30;07 years;months took part. As can be seen in **Table 1**, this age range does not introduce undue variability to the range of cognitive abilities in the WS group. This is because chronological age is rarely related to level of cognitive ability in WS (Karmiloff-Smith, 1998). WS participants were recruited via the records of the Williams Syndrome Foundation, UK. All participants with WS had received both phenotypic and genetic diagnosis by a clinician. Genetic diagnosis was via a Fluorescent *in situ* Hybridisation (FISH) test (Lenhoff et al., 1997). WS performance was compared to that of the 75 TD children assessed by Farran and Atkinson (2016) who were aged 4–7 years. Analytical comparison of patterns of performance and errors is made with reference to four separate groups of TD children: four- (*N* = 20), five- (*N* = 18), six- (*N* = 18), and 7-year-olds (*N* = 19), whilst developmental trajectory analysis treats the TD children as one group. TD participants were recruited from UK primary schools. Participant details are shown in **Table 1**.

**Table 1:** Participant details.

Group	*N*	Chronological age (years;months)		BPVS raw score		RCPM raw score		Spatial language composite score	
					
		Mean	*SD*	Mean	*SD*	Mean	*SD*	Mean	*SD*
WS	24	20;05	5;03	123.92	17.34	18.92	4.66	13.67	3.53
Four-year-olds	20	4;08	0;02	52.75	10.92	11.20	4.81	12.25	3.22
Five-year-olds	18	5;07	0;03	60.22	9.94	16.06	5.41	13.50	2.04
Six-year-olds	18	6;05	0;04	73.61	11.63	19.61	4.82	14.83	2.01
Seven-year-olds	19	7;06	0;04	84.63	11.41	5.13	19.00	15.74	2.23


**BPVS*, British Picture Vocabulary Scale; *RCPM*, Raven’s Colored Progressive Matrices.*

### Design and Procedure

Participants completed the odd-one-out task, which is our measure of spatial category representations, as well as spatial language comprehension and production tasks. Receptive vocabulary and non-verbal ability were measured using the British Picture Vocabulary Scale II (BPVS-II; Dunn et al., 1997) and Raven’s Colored Progressive Matrices (RCPM; Raven, 1993), respectively. The odd-one-out task was administered as two equivalent blocks, counterbalanced for order. BPVS and RCPM were also counterbalanced for order and interleaved with the two blocks of the odd-one-out task. The spatial language tasks were administered at the end of the session. To ensure that participants were not verbally exposed to the correct terms, the production task always preceded the comprehension task.

#### Odd-One-Out Task

The design and procedure for this task is identical to that reported by Farran and Atkinson (2016). For each trial, four images were displayed on a 15-inch laptop. Three of the four images depicted the same spatial relationship between two objects (*In, On, Under, In Front, Behind, Above, Below, Left*, or *Right*) and the fourth image displayed a different spatial relationship. Participants were instructed to use the mouse to click on the image that was the odd-one-out. The odd-one-out appeared an equal number of times in each quadrant of the display.

Reference objects were symmetrical objects such as a table or a box. Both located and reference object were broadly similar in terms of imageability (mean rating: 6.4/7; range: 5.6–6.9) and familiarity (mean rating: 4.1/5; range: 2.73–4.77). All objects had an age-of-acquisition of under 4 years (mean: 23.8 months; range 22.1–38.5 months; Morrison et al., 1997).

**FIGURE 1:**
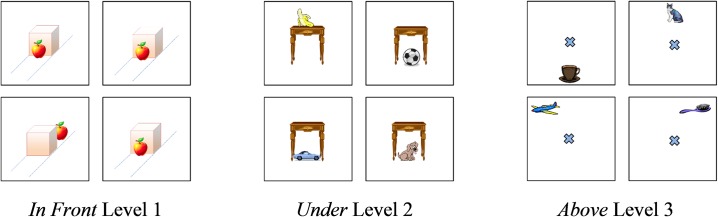
**Example of odd-one-out task stimuli**.

The three difficulty levels corresponded to the three levels of the spatial representation model. For levels 1 and 2, each image displayed a prototypical spatial relationship between the two objects (e.g., a book *on* the center of a box). For level 1 trials the two objects were the same across the four images, but the spatial relationship between the located and reference object was different for one image, the odd-one-out. For example, three images displayed an apple (located object) *in front of* a box (referent object), whilst the odd-one-out image displayed an apple *behind* of a box (**Figure 1**, Level 1). For level 2 trials, the same referent object was used (e.g., a table), but paired with a different located object for each of the four images (e.g., ball, dog, car, banana). As with level 1, three of the images showed the same spatial relationship between the two objects, whilst the fourth image displayed the opposite relationship (e.g., object *on* the table vs. object *under* the table: **Figure 1**, level 2). For level 3 trials the relationship between the located and reference object was not always prototypical. As in level 2, the four images used a different located object paired with the same reference object. Three images showed the same spatial relationship, but in contrast to previous levels one image demonstrated a prototypical example of that spatial relationship and two depicted non-prototypical examples of the same spatial relationship. The fourth, odd-one-out image, showed the opposite spatial relationship in a prototypical format [e.g., **Figure 1**, Level 3: cat *above* cross (prototypical); brush *above* cross (non-prototypical); plane *above* cross (non-prototypical); cup *below cross* (odd-one-out)]. For further examples see Farran and Atkinson (2016).

The non-prototypical spatial category examples used in level 3 were created using the following criteria. For *in*, *on*, and *under* trials, just over 50% of the located object remained *in*, *on*, or *under* the reference object, with displacement to the left and right of center for the two non-prototypical images, respectively. For *in front*, *behind*, *above, below*, *left*, and *right*, the located object was placed off the prototypical axis by just under ± 45° (based on the axial reference system, see Hayward and Tarr, 1995) such that the closest axis was still correct for that category (the two non-prototypical examples in each trial were off-axis in a positive or negative direction, respectively).

There were six practice trials, which were designed to ensure that participants understood the concept of comparing images in order to determine spatial differences. The practice trials displayed two images, each of a teddy and a box. The two images were either identical, showing the teddy and the box in the same prototypical spatial relationship, or displayed the two objects in different spatial relationships. Participants were asked: “Can you tell me whether these pictures are the same or different?.” All participants achieved 100% accuracy on the practice trials.

For experimental trials, the participant was instructed to: “Click on the picture that is different from the other pictures,” i.e., the odd-one-out. Feedback regarding accuracy was not given. There were 108 trials, presented in two equivalent blocks of 54 randomly ordered trials. In total, for each of the three difficulty levels, each spatial category type was presented four times, each time represented by different image pairs (9 spatial relationships × 3 levels × 2 trials × 2 blocks = 108 trials). Each block included five motivational screens (e.g., “Well Done!”).

The prototypical relationships used in our odd-one-out task, and the non-prototypical examples for *on* and *under* were similar to Meints et al. (2002), who demonstrated that adults rated these as typical and atypical examples of the categories, respectively. We also validated our task on ten adult participants (mean [*SD*] chronological age: 33;03 [11;08] years;months). Eight adults achieved >95% accuracy, with the remaining two participants scoring 94 and 88%. No systematic errors were present. The high performance level in the adults validates category membership for each of the nine spatial relationships employed, for all three levels.

#### Spatial Language Tasks

As with the odd-one-out task, the design and procedure for this task is identical to Farran and Atkinson (2016). Participants were shown a teddy bear and a clear plastic box. For the production trials, based on Landau and Hoffman (2005), the experimenter said: “I’m going to put the teddy in different places and when I do, I want you to look very carefully at the teddy and the box and then tell me where the teddy is. I’m going to move the teddy and the box around and then I want you to describe where the teddy is. Where is the teddy? The teddy is ____ (*In, On, Under, In Front, Behind, Above, Below, Left*, or *Right*) the box.” On rare occasions participants produced a general description (e.g., “he is *next to* the box”) and were asked once if they could be more specific.

For the comprehension trials, the experimenter said: “Now I’m going to tell you where to put the teddy. You’re going to move the teddy to different places just like I did.” The experimenter then said, for example: “Put the teddy *on* the box.”

## Results

### Verbal Ability, Non-verbal Ability, and Spatial Language Measures

British Picture Vocabulary Scale raw score, RCPM raw score, and spatial language composite score (the sum of comprehension and production performance) for each group are shown in **Table 1**. Three ANOVAs were carried out to compare the performance of the WS group to that of the four TD groups, for each dependent variable. Each ANOVA had Group (five levels: WS, 4-, 5-, 6-, and 7-year-olds) as the between participants factor and either BPVS raw score, RCPM raw score or spatial language composite score as the dependent variable. Three individuals with WS did not want to complete the spatial language task and so analyses with this task have a reduced N (WS *N* = 21). There were significant effects of Group for each ANOVA (*p* < 0.05 for each). Of interest here are *post hoc* Tukey tests comparing the performance of the WS group against that of each TD group. As expected, BPVS scores for the WS group were significantly higher than those from all TD groups (*p* < 0.001 for all comparisons), whilst the RCPM scores of the WS group were significantly higher than those of 4-year-old TD children (*p* < 0.001), at the same level as those of the 5- and 6-year-old TD groups (*p* > 0.05 for both), but significantly below the scores of the 7-year-old TD children (*p* = 0.013). This reflects the typical WS profile of stronger verbal ability in the face of weak spatial abilities. Spatial language score of the WS group did not differ significantly from any of the TD groups (*p* > 0.05; the main effect of Group reflected higher scores among TD children at 6 and 7 years than at 4 years, *p* < 0.05 for both). The percentages of individuals with WS and TD children (4–7 years as one group) who were able to comprehend and produce each spatial term are shown in **Table 2** (for percentages for individual TD age groups, see Farran and Atkinson, 2016).

**Table 2:** Percentage of Williams syndrome (WS) and typically developing (TD: 4–7 years) participants able to comprehend and produce each spatial language term.

Spatial term	Group	TD (4–7 years)	WS
In	Comprehension	100	100
	Production	100	100
On	Comprehension	100	100
	Production	96.0	90.5
Under	Comprehension	100	100
	Production	77.3	90.5
In front	Comprehension	100	90.5
	Production	85.3	66.7
Behind	Comprehension	100	90.5
	Production	77.3	52.4
Above	Comprehension	93.3	71.4
	Production	29.3	42.9
Below	Comprehension	82.7	61.9
	Production	9.3	28.6
Left	Comprehension	70.7	76.2
	Production	56.0	61.9
Right	Comprehension	70.7	81.0
	Production	57.3	61.9


### Odd-One-Out Task

#### Profiles of Performance across Spatial Categories and Levels

**FIGURE 2:**
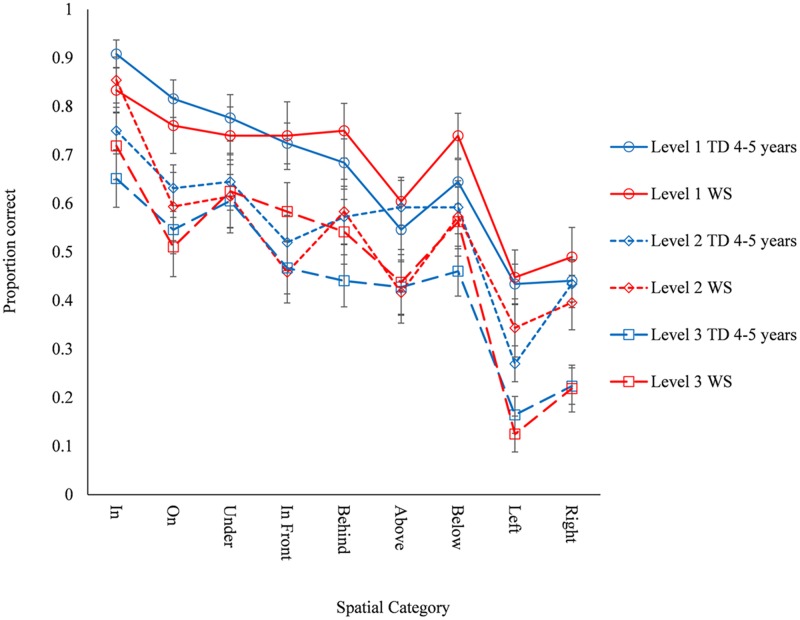
**The development of spatial category understanding on the odd-one-out task across level and group**.

Full exploration of typical development on the odd-one-out task is reported in Farran and Atkinson (2016). ANOVA with a between participant factor of Group (five levels: WS, 4-, 5-, 6-, and 7-year-olds) and within-participant factors of Spatial Category (nine levels: *in, on, under, in front, behind, above, below, left, right*) and Level (three levels) was carried out to determine which age group the WS group best resembled at an overall task level. As anticipated, there was a significant main effect of Group, *F*(4,94) = 23.136, *p* < 0.001, $\eta_{p}^{2}$ = 0.496. Tukey *post hoc* comparisons determined that the WS group performed similarly to the 4-year-old (*p* = 0.159) and 5-year-old (*p* = 0.299) groups, but below the general performance level of the 6-year-olds (*p* < 0.001) or 7-year-olds (*p* < 0.001). Given that there are developmental differences in the typical population across both Spatial Category and Level (Farran and Atkinson, 2016), the ANOVA was run again, this time treating the 4- and 5-year-olds as a single TD group who are matched at a group level for overall task performance, for comparison against the WS group. ANOVA with a between participant factor of Group (two levels: WS, TD) and within-participant factors of Spatial Category (nine levels) and Level (three levels) was carried out (**Figure 2**). Spatial categories were entered in the documented order of acquisition (*in, on, under, in front and behind, above and below, left and right*), and the significant main effect of Spatial Category was best described as linear, reported as a linear contrast *F*(1,60) = 174.276, *p* < 0.001, $\eta_{p}^{2}$ = 744. The main effect of Level was also best described as linear, *F*(1,60) = 160.334, *p* < 0.001, $\eta_{p}^{2}$ = 728. By design the main effect of Group was not significant (*F* < 1). There was also an interaction between Spatial Category and Level, *F*(16,960) = 2.296, *p* = 0.003, $\eta_{p}^{2}$ = 0.037. Importantly, there were no significant interactions with Group: Spatial Category by Group, *F* < 1; Level by Group, *F*(2,120) = 1.780, *p* = 0.173, $\eta_{p}^{2}$ = 0.029, Spatial Category by Level by Group, *F*(16,960) = 1.451, *p* = 0.111, $\eta_{p}^{2}$ = 0.024, which demonstrates that the pattern of spatial category representation in the WS group was typical, albeit at the level of TD 4- and 5-year-olds. Exploration of the Spatial Category by Level interaction demonstrated that for all spatial categories, linear progression of poorer performance with increasing difficulty best described the effect of level. This was supported by pairwise comparisons, which also further demonstrated three patterns: level 1 > level 2 > level 3 (*on, left)*; level 1 > level 2 = level 3 (*under, in front, behind, below)*; level 1 = level 2 > level 3 (*in, above, right)*.

#### Developmental Trajectory Analysis

We employed the developmental trajectory approach (Thomas et al., 2009) to explore the mechanistic development of spatial category representations. Using this approach, one can observe how variance in performance on a task relates to variance in the cognitive maturation of mechanisms that are thought to contribute to task performance. Here we took a theoretically driven approach that the odd-one-out task would be influenced by a participant’s non-verbal ability, but also that participants might place some reliance on their spatial language ability to complete the odd-one-out task. Performance on the odd-one-out task was collapsed across level and spatial category to produce one odd-one-out task variable of overall proportion correct. Because we are interested in developmental variability, the whole TD group (*N* = 75) was used in these analyses, for comparison with the WS group. Based on the developmental trajectory approach (Thomas et al., 2009), ANCOVAs were employed. For this method, the mechanistic measure of cognitive ability is employed as the covariate, which enables one to characterize the linear developmental trajectory of performance on the experimental task, as driven by individual differences in underlying cognitive ability. We ran two analyses to explore the mechanistic input of non-verbal ability [measured using RCPM (Raven, 1993)] and spatial language on performance on the odd-one-out task, respectively. One can think of this as exploring group difference in odd-one-out performance in relation to non-verbal ‘mental age (MA)’ or spatial language ‘MA.’ Thus RCPM raw score or spatial language score were employed as the covariates for each ANCOVA, respectively.

Raven’s Colored Progressive Matrices and spatial language scores were rescaled such that the analysis reflected the intercept at the lowest RCPM score of the WS group. This does not change the analysis, but aids interpretation.

**FIGURE 3:**
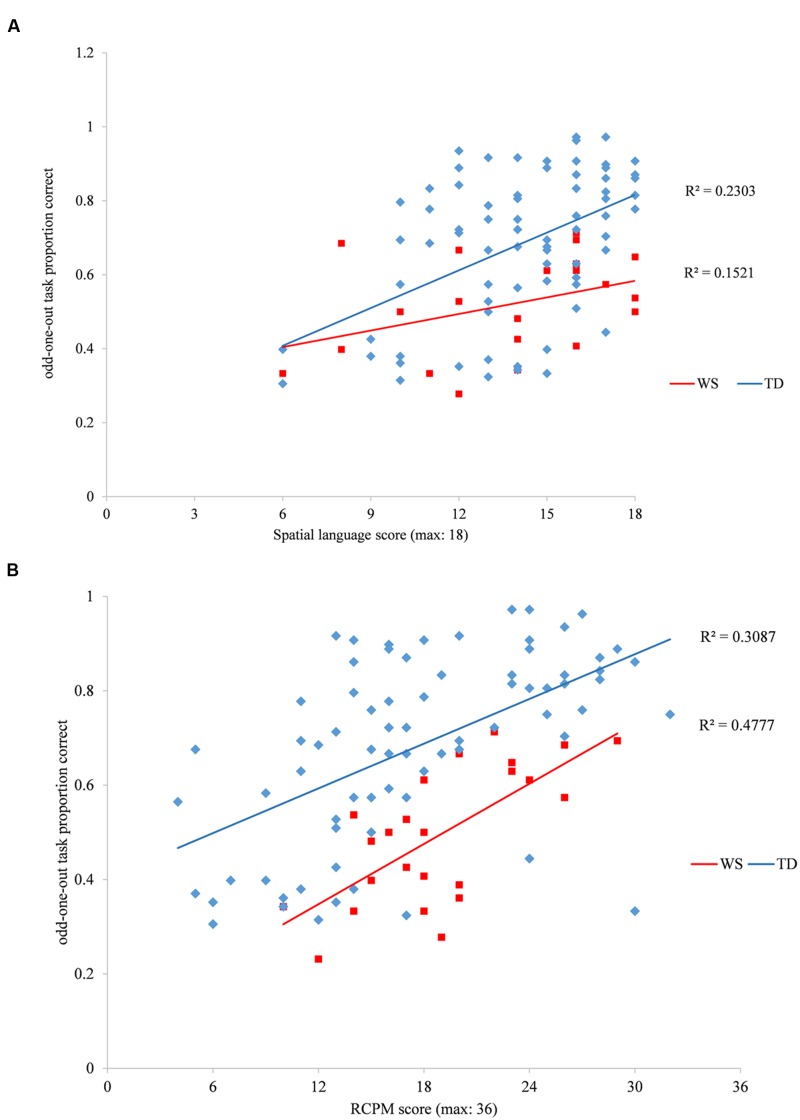
**(A)** Williams syndrome (WS) and typically developing (TD) trajectories of odd-one-out performance when plotted against spatial language composite (comprehension and production) score. **(B)** WS and TD trajectories of odd-one-out performance when plotted against Ravens Coloured Progressive Matrices (RCPM) score.

ANCOVA of odd-one-out proportion correct, with Group as a between participant factor (WS, TD) and RCPM score as a covariate revealed a relationship between RCPM score and odd-one-out performance, *F*(1,95) = 25.975, *p* < 0.001, $\eta_{p}^{2}$ = 0.215. This was true for each group individually (TD: *r*^2^ = 0.309, *p* < 0.001; WS: *r*^2^ = 0.478, *p* < 0.001). The developmental trajectories of odd-one-out performance differed significantly at the intercept, such that the WS group had consistently lower odd-one-out scores than the TD group at the lowest RCPM scores, *F*(1,95) = 12.345, *p* = 0.001, $\eta_{p}^{2}$ = 0.115. Furthermore, the slopes of the trajectories (determined by the RCPM by Group interaction) were equivalent between groups (*F* < 1). That is, the pattern of lower odd-out-out scores in the WS group compared to the TD group was consistent across the range of RCPM scores, indicative of ‘delayed’ development, relative to non-verbal MA (**Figure 3A**). ANCOVA of odd-one-out proportion correct, with Group as a between participant factor (WS, TD) and spatial language score as a covariate demonstrated a strong overall relationship between spatial language and odd-one-out task performance, *F*(1,92) = 15.301, *p* < 0.001, $\eta_{p}^{2}$ = 0.143. This relationship was significant for the TD group and marginal for the WS group (TD: *r*^2^ = 0.230, *p* < 0.001; WS: *r*^2^ = 0.152, *p* = 0.08), although note that the correlation coefficients were comparable (Fisher’s r-to-z transformation, *z* = 0.0420, *p* = 0.674). The ANCOVA demonstrated a similar intercept between the groups (*F* < 1), and similar rates of development (spatial language by Group interaction): *F*(1,92) = 2.334, *p* = 0.130, $\eta_{p}^{2}$ = 0.025 (**Figure 3B**), indicative of typical development in the WS group for their (low) spatial language MA.

#### Error Analysis

Analysis thus far has suggested that spatial category representation in WS is largely typical, albeit at the level of young TD children. We also compared the errors made by both the TD and WS groups on level 3 trials. In levels 1 and 2, the three images that showed the same category were spatially identical and so error analysis would not be fruitful. In level 3 trials participants were required to categorize both prototypical and non-prototypical examples of a category as the same category in order to locate the odd-one-out. If spatial category representations are structured differently between groups, it is possible that the WS and TD groups differ in the kinds of errors that they make at level 3. There were two kinds of errors for level 3 trials; choosing the image that displayed a prototypical example of the spatial relationship or choosing one of the two images that displayed a non-prototypical example of the spatial relationship.

Because ANOVA requires independence of scores, it would not be appropriate to compare the two error types in a single analysis. Instead a proportion statistic was created based on the proportion of overall errors in which the participant chose a non-prototypical distracter divided by the total number of errors made. Given that within a single trial there were twice as many non-prototypical examples as prototypical examples of the category, a proportion of 0.667 would indicate a guess response. Mean (SD) proportion of non-prototypical errors made were as follows: WS: 0.527 (0.112); TD 4 years: 0.723 (0.121); TD 5 years: 0.659 (0.144); TD 6 years: 0.705 (0.246); TD 7 years: 0.638 (0.258). One sample *t*-tests of each group against 0.667 demonstrated chance level responding in all TD groups (*p* > 0.05 for all). The WS group were less likely than chance to choose a non-prototypical category member as the odd-one-out (*p* < 0.001). ANOVA of the proportion of non-prototypical errors, with Group as a between participant factor (five levels: WS, 4-, 5-, 6-, and 7-year-olds) demonstrated a significant group difference, *F*(4,93) = 3.722, *p* = 0.007, $\eta_{p}^{2}$ = 0.138. Tukey *post hoc* tests revealed that this was due to a bias away from non-prototypical errors (toward prototypical errors) in the WS group relative to the TD 4-year-olds (*p* = 0.007) and TD 6-year-olds (*p* = 0.023), but not the TD 5-year-olds (*p* = 0.164) or TD 7-year-olds (*p* = 0.304). All comparisons across TD groups were non-significant (*p* > 0.05). It is unlikely that this related to the older age range of the WS group because chronological age did not correlate with proportion of non-prototypical errors for either the WS group (*r* = -0.122, *p* = 0.579) or the TD group [treated as one continuous group (*N* = 75); *r* = -0.120, *p* = 0.305).

## Discussion

The predominant aim of the current study was to characterize spatial category representations in WS across nine spatial categories with reference to the spatial category representation model (Farran and Atkinson, 2016). We demonstrated that spatial category representations in this sample of 12- to 30-year-olds with WS are at the level of TD 4- to 5-year-old children. Furthermore, where previous studies have explored spatial category representations for up to four spatial categories, we have demonstrated that spatial category representations are impaired in WS across nine spatial categories. This suggests that an impairment in spatial category representations in WS is universal across all spatial categories, rather than a specific deficit to particular spatial category representations. The level of spatial category representations observed here for the WS group contrasts to receptive vocabulary scores that are higher than that of TD 7-year-olds. This contrast reflects the WS cognitive profile of impaired visuo-spatial ability compared to a relative strength in verbal ability (e.g., Udwin and Yule, 1991). Level of performance on spatial category representations in WS is similar to that reported on block construction tasks (Farran and Formby, 2012). This supports the notion that impaired spatial category representation could be a contributing factor to the hallmark impairment in visuo-spatial construction and drawing ability in WS (Mervis et al., 1999).

Laing and Jarrold (2007) attributed poor performance on their spatial picture matching task to impaired spatial mental models in WS. The deficit observed in the current study supports this suggestion because a mental model of the characteristics of a spatial category is vital for the development of a stable representation of that spatial category. Poor spatial category representation, as observed here would dictate a difficulty with visuo-spatial construction and drawing in a number of ways. First, the ability to determine the relationship between one part of an image and another would be impaired, and thus explain the reported lack of global cohesion in WS. Equally, it seems logical that the problem discriminating between block faces, for example a block face with white at the top and red at the bottom from a block face with the opposite pattern (Hoffman et al., 2003; Farran and Jarrold, 2004), is attributable to poor spatial category representations. Finally, if spatial category representations are fragile, then checking a part-finished solution against the model image for accuracy in the reproduction of spatial relations would be of limited benefit. This could explain why this strategy is used sparingly in WS (Hoffman et al., 2003; Hudson and Farran, 2013). Future research could directly explore the impact of poor spatial category representation on visuo-spatial construction and drawing ability in WS.

The current findings support the two previous studies that report impaired spatial relationship understanding in WS. Landau and Hoffman (2005) demonstrated narrow category membership for the categories of *above*, *below*, *left*, and *right* in WS compared to MA matched TD children (Landau and Hoffman, 2005), whilst Farran and Jarrold (2005) reported atypical category boundaries for *above* vs. *below* relative to TD children and adults, also suggestive of narrow category membership in WS. According to the spatial category representation model (Farran and Atkinson, 2016), broad category membership, i.e., understanding that both prototypical and non-prototypical examples of spatial categories belong to the same category, is the most sophisticated stage in spatial category representation. In the current study, this was investigated by level 3 trials specifically. Whilst level 3 performance was limited in WS, thus supporting previous reports of narrow category membership, this was in the context of impaired spatial category representations at all three levels. Thus, we can conclude that the deficit in spatial category representations in WS extends beyond the presence of narrow category membership, but represents a fundamental deficit across all three levels of the spatial category representation model, rigid (level 1), abstract (level 2), and broad category membership (level 3; Farran and Atkinson, 2016). Equally, given that the patterns of ability across levels are typical in WS this also suggests that WS performance on this task represents delayed development as opposed to a specific deficit at level 3 or any other deviance in spatial category representation. A further aim of the current study was to probe level 3 understanding by analyzing patterns of error responses. In contrast to evidence so far of simple delay, this analysis showed a pattern of error responses in the WS group that was not observed in the TD groups. That is, whilst the TD children guessed when they did not know the answer, the participants with WS were more likely than chance to pick the prototypical category member when they made an error. This difference must be considered with caution, however, as the proportion errors made by the WS group which involved choosing a non-prototypical category member, was not significantly different from that of the TD 5- or 7-year-olds. It is possible that the WS group had surmised from previous trials (be they levels 1 and 2 where all images displayed prototypical relationships, or level 3 where two of the four images displayed prototypical relationships) that the correct answer (the odd-one-out) was always a prototypical category member and so, when they were uncertain, a best guess was to pick one of the two images that showed prototypical category examples. Why this would have become apparent to the WS group and not the TD group, however, is not apparent; as such this explanation is difficult to support. Another possibility relates to the difficulty with orientation discrimination observed in WS (Farran and Jarrold, 2004; Farran, 2006). For level 3 trials, all of the images that displayed non-prototypical category exemplars were non-symmetrical, whereas the odd-one-out image and its opposite were symmetrical. Perhaps the individuals with WS found it relatively difficult to discriminate between the two non-symmetrical images on account of the oblique orientations between the locations of the located and referent objects, and were thus less likely to pick one of these as the odd-one-out, and more likely to pick one of the symmetrical images (one of which was the odd-one-out, whilst the other counted as an error) simply because they were better able to distinguish it from the other images.

In the current study, in order to maintain equity across our nine spatial categories, level 3 trials only depicted one type of non-prototypical category example; off-axis representations. Another type of non-prototypical category example would be to manipulate the distance between the object and referent, but this would not have been possible to manipulate across all categories (for example, *on* requires contact and so it is not possible to vary the distance between object and referent) and so was not employed. Our finding that WS performance is at the level of 4- to 5-year-olds for off-axis examples supports Landau and Hoffman’s (2005) claims. Further research is necessary to determine the developmental level reached by individuals with WS for category members that vary by distance between the object and referent, but evidence from Landau and Hoffman (2005) suggest similar or lower impairment in WS relative to off-axis examples. Equally, some categories have more than one sub-type such as support and tight fit versions of *on* (a book *on* a table vs. a ring *on* a finger). These also merit research with individuals with WS.

Despite a developmentally low level of spatial category representations in WS, with the exception of the error analysis, the notable finding that pervades the analyses in this study is that performance across the various components of the odd-one-out task matched the pattern seen among TD children. That is, both WS and TD groups demonstrated increasingly sophisticated spatial category representations in line with the documented order of acquisition of spatial category perceptual development in infancy. With reference to the spatial category representation model (Farran and Atkinson, 2016), participants reached a higher level of understanding for categories acquired earlier in development such as *in* and *on*, relative to later-acquired categories such as *left* and *right*. This was true of both the WS and the TD 4- and 5-year old group assessed here.

Another aim of the current study was to determine the extent to which (a) spatial language performance and (b) non-verbal cognitive ability, contribute to spatial category representations in WS. Developmental trajectories demonstrated that with reference to non-verbal MA (RCPM score), spatial category representation showed the same rate of development as the TD children, albeit at a consistently lower level than TD children of the same non-verbal MA. Note that it is not unusual to demonstrate peaks and troughs in ability even within a domain in WS; within the non-verbal domain, performance on the RCPM (our measure of non-verbal MA) is typically significantly stronger that a number of other non-verbal tasks, including block construction in individuals with WS (Farran et al., 1999). This delayed developmental trajectory, therefore, further demonstrates that spatial category representations are a particular weakness in WS, even within the context of their general impairment in non-verbal ability.

Laing and Jarrold (2007) report a difficulty in WS in creating a spatial mental model and mapping spatial language words to the model. The odd-one-out task requires participants to categorically group three images together according to a common spatial category in order to determine the odd-one-out. This arguably requires the individual to have a mental model of that category. Perhaps the delayed developmental trajectory with reference to non-verbal ability reflects a limited ability in WS to create mental models (a non-verbal skill).

Developmental trajectory analyses using spatial language as a MA measure demonstrated similar trajectories for the TD and WS groups with reference to level of ability and rate of development. Landau and Hoffman (2005) report a relationship between spatial language and spatial category representation in WS, but were not able to investigate this statistically and so it is not possible to determine the strength of this relationship. Laing and Jarrold (2007) report that individuals with WS might have difficulty mapping spatial language words to their impoverished mental models. The current study suggests that both individuals with WS and TD children can employ spatial language to similar success to bolster performance on the odd-one-out task. If spatial mental models are impaired, particularly in WS, perhaps this reflects the verbal labeling of each of the images rather than the more sophisticated technique of mapping spatial language onto a mental model of a category. The use of verbal labeling is consistent with previous reports that individuals with WS benefit from verbal coding on non-verbal tasks (Farran et al., 2010).

Note that spatial language only accounted for a small amount of variance in odd-one-out task performance (23% for the TD group and a non-significant 15% for the WS group) relative to the percentage of variance accounted for by non-verbal (RCPM) ability (31% for the TD group and 48% for the WS group) so it is unlikely that the groups were reliant on spatial language to complete the task, particularly as participants did not have all of the spatial language terms available to them (Farran and O’Leary, 2016). Rather, it suggests that spatial language could be used as a facilitator if available. The task was deliberately designed to be non-verbal in nature. Spatial category representations could be accessed non-linguistically, or linguistically, dependent on the resources available to the individual (Casasola, 2008 for a similar discussion of the emergence of spatial category representation in infancy). The trajectory analyses suggest that for both the TD and WS group, the odd-one-out task drew on both non-linguistic and linguistic processing. The smaller effect sizes for the input of spatial language could indicate less reliance on linguistic (spatial language) input than non-linguistic input (RCPM) for both groups.

The spatial language abilities of the WS group did not differ from 4- to 7-year-old TD children. Because of this comparable level of performance, we cannot be sure of the exact upper and lower MA range of spatial language ability of the WS group. However, there was developmental improvement in spatial language ability between 4 and 7 years in the TD group and so it is unlikely that the spatial language abilities of the WS group spanned much beyond the TD age range tested. This contrasts to BPVS scores which were higher in the WS group than all of the TD age groups tested here. This contrast demonstrates that despite a relative strength in verbal ability in WS, this does not encompass spatial language ability. This is consistent with previous assessments of spatial language in WS (Phillips et al., 2004; Landau and Hoffman, 2005; Laing and Jarrold, 2007; Heinze et al., 2014). This impairment demonstrates a cross-domain interaction; spatial language ability, although part of the verbal domain, is more in line with spatial cognition than verbal cognition in WS. This supports the neuroconstructivist approach to development that development is the result of a dynamic interactive process, and that early development in infancy can give rise to cascading impacts on the development of later emerging skills (Karmiloff-Smith, 1998; Farran and Karmiloff-Smith, 2012). Here, it is likely that small deficits in the visuo-spatial domain gave rise to deficits in both spatial category representations (visuo-spatial domain) and spatial language (verbal domain).

## Conclusion

Spatial category representations in WS are at the level of TD 4- to 5-year-olds, but show a typical profile of category proficiency and conform to the spatial category representation model (Farran and Atkinson, 2016). We suggest that this deficit contributes to the hallmark impairment in visuo-spatial construction and drawing observed in WS.

## Author Contributions

EF and LA designed the task. LA collected the data from the typically developing children and data from 12 of the participants with Williams syndrome. HB collected data from 12 of the participants with Williams syndrome and commented on drafts of the manuscript. EF wrote the manuscript.

## Conflict of Interest Statement

The authors declare that the research was conducted in the absence of any commercial or financial relationships that could be construed as a potential conflict of interest.
